# Prognostic significance and mechanisms of CXCL genes in clear cell renal cell carcinoma

**DOI:** 10.18632/aging.204922

**Published:** 2023-08-03

**Authors:** Junwen Shen, Rongjiang Wang, Yu Chen, Zhihai Fang, Jianer Tang, Jianxiang Yao, Jianguo Gao, Xiaonong Chen, Xinli Shi

**Affiliations:** 1 The Department of Urology, The First Affiliated Hospital of Huzhou Normal College, Huzhou, Zhejiang 31300, China; 2 Huzhou Key Laboratory of Precise Diagnosis and Treatment of Urinary Tumors, Huzhou, Zhejiang 31300, China

**Keywords:** CXCL genes, clear cell renal cell carcinoma, neutrophil recruitment, TAN phenotype, bioinformatics analysis

## Abstract

This study aimed to investigate the clinical significance, biological functions, and underlying mechanisms of CXCL genes in clear cell renal cell carcinoma (ccRcc) based on patient datasets and pan-cancer analysis. The interaction between CXCL genes in ccRcc and immune components, particularly in relation to neutrophil recruitment and polarization mechanisms, was also evaluated. Furthermore, a risk score was developed using a signature for neutrophil polarization. The role of CXCL2 was assessed through *in vitro* experiments. Results showed that five CXCL genes (CXCL 2, 5, 9, 10, and 11) were upregulated in renal cancer tissue, while seven genes (CXCL 1, 2, 3, 5, 8, 13, and 14) significantly impacted patient survival. Moreover, CXCL 1, 5, and 13 affected progression-free survival. Besides, differences in mRNA expression and immune components affected renal cancer outcomes. Furthermore, three pairs of CXCL gene-immune cell interactions (CXCL13-CD8+ T cells, CXCL9/10-M1 cells, CXCL1/2/3/8-neutrophils) were identified through single-cell and pan-cancer analysis. A TAN risk score with prognostic value for KIRC patients was constructed using 11 genes and a TAN signature. Neutrophil polarization significantly impacted survival. Notably, CXCL2 was involved in neutrophil recruitment and polarization, thus promoting ccRcc progression. In conclusion, seven prognostic CXCL genes (CXCL 1/2/3/5/8/13/14) for ccRcc patients and three pairs of CXCL gene-immune cell interactions were identified. Furthermore, results showed that CXCL 2 promotes ccRcc progression through neutrophil recruitment and polarization.

## INTRODUCTION

Cytokines mediate cellular communication in the tumor microenvironment (TME) [1]. Cytokines and their receptors are abundant in the TME of cancer, which promote and inhibit cancer growth. Some cytokines, such as IL-2, IFNα, and IFN γ, can help fight against tumors in the TME. Abnormal generation of cytokines by cancer cells, the immune system, and the stroma cells promotes the development and resistance of cancer [2]. Therefore, further understanding of cytokines and their impact on human cancer is crucial.

Some CXCL family genes, including CXCL8, are related to cancer progression [3]. In this study, the function and mechanism of CXCL genes in renal clear cell carcinoma (ccRcc), especially their prognostic significance and interaction with immune components, were analyzed based on multiple data sources (mRNA datasets and pan-cancer data). The results were analyzed using multiple dimensions (bulk RNA- sequencing and single-cell RNA-sequencing evaluations). Furthermore, neutrophil polarization towards a pro-tumor state promoted ccRcc development mainly mediated by CXCL2-CXCR1. Bioinformatic analysis and *in vitro* experiments showed that CXCL2 promotes ccRcc through many mechanisms.

## MATERIALS AND METHODS

### Data sources

Four types of publicly available data sources were used in this study, including TCGA-KIRC dataset, ICGC website data, GEO datasets, and clinical trial data. The TCGA-KIRC dataset was the primary data source, where mRNA expression, gene function prediction, immune component, prognostic value, and other dimensions of CXCLs genes were analyzed. Additionally, a multi-dimensional Pan-cancer analysis was conducted based on 33 TCGA cancer datasets. The TCGA data were obtained from the TCGA website (https://www.cancer.gov/about-nci/organization/ccg/research/structural-genomics/tcga) [4]. Gene expression and clinical information were also obtained from the GEO dataset website (https://www.ncbi.nlm.nih.gov/gds/) [5]. Data from renal cancer collected by the International Cancer Genome Consortium (ICGC) were obtained from the ICGC website (https://dcc.icgc.org/) [6]. Furthermore, a clinical trial, the Checkmate Trial (CM Trial) [7], analyzing the effect of PD-1 blockade on advanced clear renal cell carcinoma patients, was included in this research. The gene expression, clinical information, and other data from this trial are available in the Supplementary Materials of the research paper.

### Prognostic analysis

Prognostic analysis was conducted after collecting RNA sequencing expression profiles and corresponding clinical information from the original datasets. The prognostic value of single gene expression or a specific score was determined using Kaplan-Meier (KM) method. The time-dependent prognostic value or comparison of the prognostic value of different indices was achieved using receiver operating characteristic (ROC) curve. A Cox Proportional Hazards Regression analysis was also conducted.

### Unsupervised cluster analysis

Consensus Cluster Plus (v1.54.0), an R package, was used for the unsupervised cluster analysis [8]. The maximum number of clusters was set to 6, and 80% of the total samples were selected for analysis. The process was repeated 100 times.

### Immunity analysis

Immune components and related scores were analyzed using immuneeconv R package [9]. This package integrates six algorithms, including TIMER, xCELL, MCP-counter, CIBERSORT, EPIC, and quanTIseq, and provides direct evaluation and analysis of eight immune checkpoint expressions.

### Mutation analysis

The mutation data were downloaded and visualized using the maftools R package. The mutation status and correlation of CXCL family genes were analyzed via cBioPortal (https://www.cbioportal.org/) [10].

### Single cell analysis

Two GEO datasets of single cells from renal cancer (GSE171306 and GSE121636) were included for single cell analysis. An object was developed using the R package “Seurat” [11] we created, then cells with low quality were filtered out. Standard data preprocessing, involving excluding genes that were detected in fewer than three cells and cells with fewer than 200 genes, was performed. The library size of each cell was normalized by scaling UMI counts with a factor of 10000. The data were log-transformed, then corrected for variation using the scale data function in Seurat. The corrected and normalized data were used for standard analysis following the Seurat R package guidelines. The top 1000 variable genes were selected for Principal Component Analysis (PCA) while the top ten components were used for UMAP visualization and clustering.

### TAN-related differentially expressed gene signature

Tumor-associated central granulocyte (TAN) is a crucial survival index in the TME. TAN polarization can promote or inhibit cancer. In this study, markers (Supplementary Table 1) were collected from two TAN subtypes (N2 subtype (pro-tumoral) and N1 subtype (anti-tumoral)) [12]. An unsupervised cluster analysis was then performed based on the KIRC dataset using TAN subtype markers and identified groups 1/2 as TAN N1 and TAN N2 models, respectively. The differentially expressed genes (DEGs) between the two groups were detected using the R package limma at *P* < 0.001. TAN-related DEGs were detected using a Cox proportional hazards regression analysis (*P* < 0.01). A TAN-related signature was developed using the Least Absolute Shrinkage and Selection Operator (LASSO) regression algorithm [13]. LASSO and glmnet package in R were used for feature selection and cross-validation (10 times), respectively.

A new signature containing TAN-related DEGs was predicted by calculating the risk score of each sample as follows:


Risk Score=∑i (Coef i×Exp Gene)

where “Coef” and “Exp Gene” represent the non-zero regression coefficients obtained through the LASSO regression analysis and the expression values of the genes, respectively.

### Functional enrichment analysis

Enrichment analysis is widely used to explore gene function and associated high-level genomic information. In this study, enrichment evaluation was conducted using Gene Ontology (GO) and Kyoto Encyclopedia of Genes and Genomes (KEGG) via R’s Cluster Profiler package [14]. Additionally, Gene Set Variation Analysis (GSVA) was used to assess the correlation between genes and pathway scores via the “GSVA” R package [15].

### Prediction of molecular docking potential

AutodockVina 1.2.2 [16], a computational protein-ligand docking software, was used to study the binding strengths and interactions between CXCL2 and CXCR1/2. The molecular structures of CXCL2 and CXCR1/2 were obtained from PubChem compound [17] (https://pubchem.ncbi.nlm.nih.gov/), while their 3D coordinates were downloaded from the PDB (http://www.rcsb.org/) [18]. The protein and molecular files were converted into the PDBQT format, which involved excluding water molecules and adding polar hydrogen atoms. The grid box was centered to encompass the protein domain and provide room for molecular movement.

### *In vitro* experiment

#### Cell line

Human kidney cell line (293T) and a ccRcc cell line (Caki-1) were obtained from the Authenticated Cell Culture Compilation of China to investigate the amounts of CXCL in non-cancerous and cancerous kidney cells. The cell lines were treated following the guidelines of the Authenticated Cell Culture Compilation of China.

### CXCL mRNA and protein analysis

CXCL expression levels between non-cancerous and cancerous kidney cells were compared using Q-PCR, western blot (WB), and Elisa assay, as previously described. TOYOBO ReverTra Ace was used for quantitative RT-PCR with TOYOBO primers. Primary antisera directed against the GAPDH and CXCL genes, and secondary antisera from Boiss (Beijing, China), were utilized in the WB analysis. GAPDH was utilized as an internal control. Image J program was utilized to analyze western blot images. Hilink’s (Nanchang, China) CXCL2 Elisa assay kit was used to measure CXCL2 protein levels.

### Cell transfection

Three siRNAs targeting CXCL2 were obtained from General Biology Company, China, for transfection analysis. Quantitative PCR was employed to evaluate the efficiency of transfecting cells. The siRNA with the lowest CXCL2 expression was chosen for functional study.

### CCK-8 and Transwell assays

The CCK-8 assay was utilized to assess the proliferation of renal carcinoma cells via Dojindo’s ECC Kit-8 (Enhanced Cell Counting Kit-8, Japan), following the manufacturer’s protocols. The migratory and invasive capabilities of kidney carcinoma cell lines were evaluated using Transwell assays.

### Sunitinib *in vitro* resistance analysis

Sunitinib maleate was obtained from MedChemExpress, USA (product ID: HY-10255A). The Caki-1 cell line was treated with increasing doses of sunitinib. A CCK-8 assay was conducted after sunitinib treatment for over 48 hours to analyze cell survivability. GraphPad Prism software was used to draw the medication sensitivity curves and determine IC50 values.

### Statistical analysis

R rendition 4.0.3 and GraphPad Prism rendition 9.4 were used for all data analysis. *P* < 0.05 was considered statistically significant.

### Availability of data and materials

The original data used in this study are included in the article. The data were obtained from public databases.

## RESULTS

### Analysis of CXCL mRNA expression in multi-renal cancer datasets

**Figure 1:**
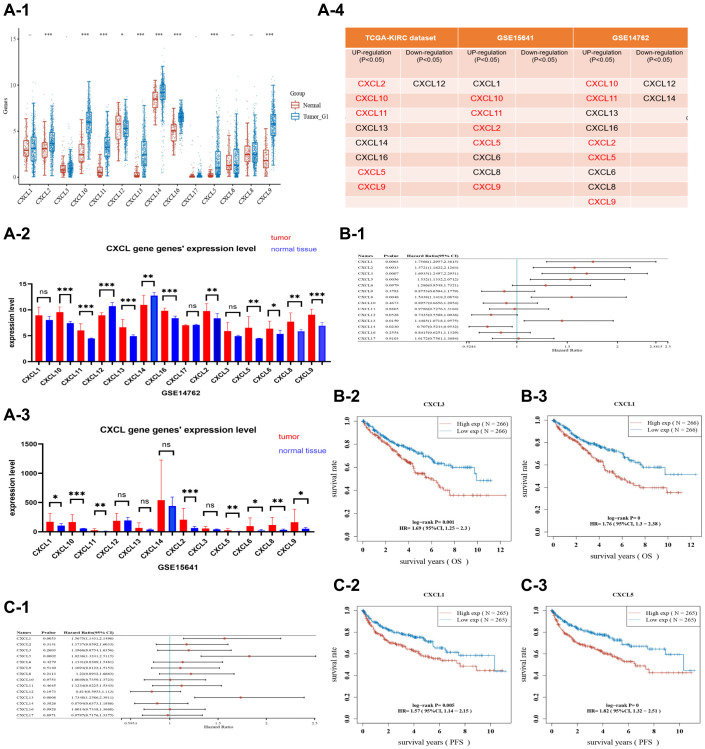
A plot showing the expression of CXCL genes in three datasets, TCGA-KIRC (**A-1**), GSE14762 (**A-2**), and GSE15641 (**A-3**). The expression of selected CXCL genes (CXCL 2/5/9/10/11) was upregulated in cancer tissues from the three datasets (**A-4**). In the TCGA-KIRC dataset, the OS and PFS of patients were analyzed. Selected CXCL genes (CXCL 1/2/3/5/8/13/14) were found to influence the prognosis of OS as shown in the forest plot (**B-1**). KM analysis was also performed on CXCL3 (**B-2**) and CXCL1 (**B-3**), with CXCL3 showing a *P*-value of 0.001 and CXCL1 showing a *P*-value of < 0.001. In the FPS analysis, CXCL 1/5/13 were associated with prognostic outcomes (**C-1**), and KM analysis was performed on CXCL1 and CXCL5, with CXCL1 showing a *P*-value of 0.005 and CXCL5 showing a *P*-value of < 0.001 (**C-2**, **C-3**).

**Figure 2:**
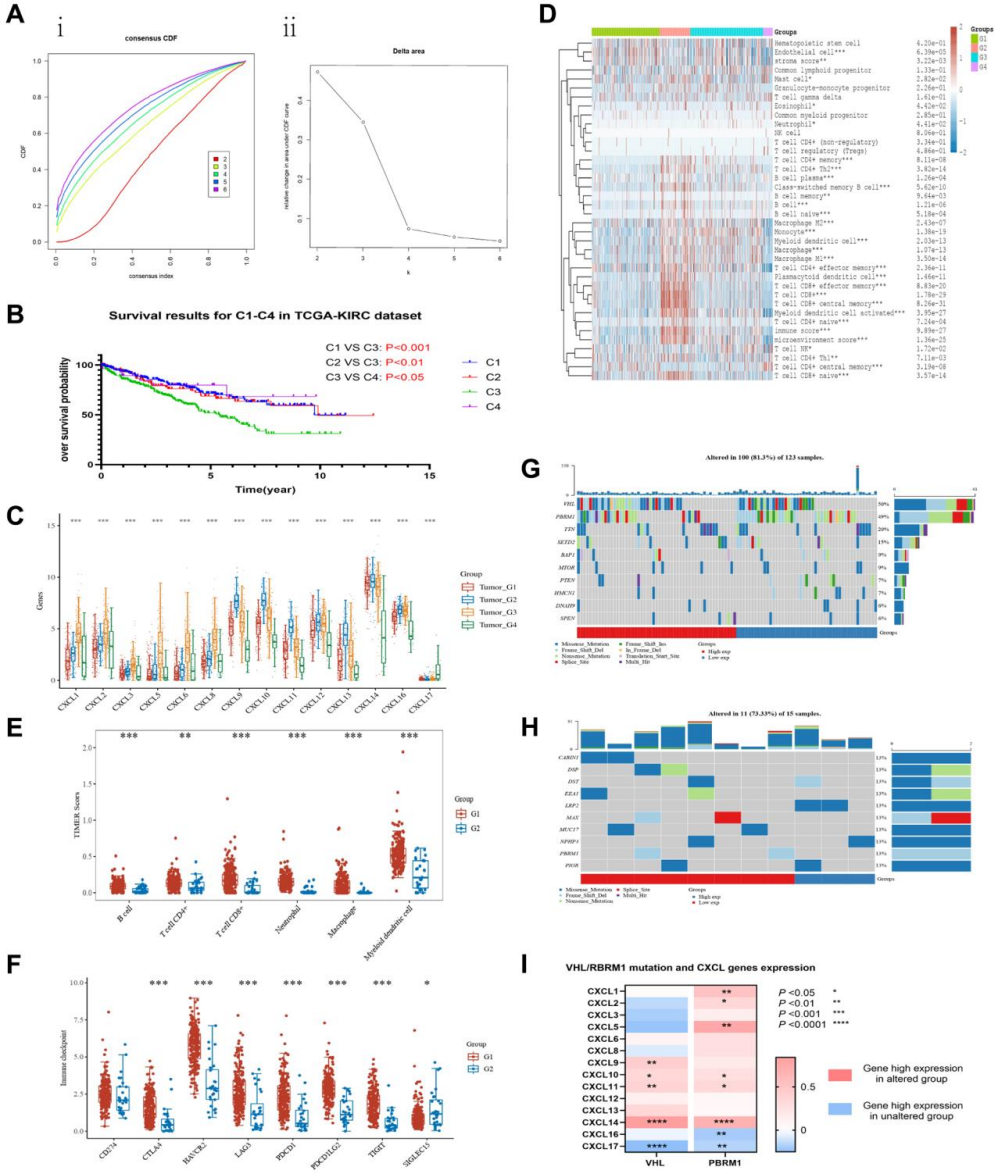
**Unsupervised cluster analysis was performed on the TCGA-KIRC dataset to group patients based on the expression level of CXCL genes.** The results show the distribution of four clusters, CDF plot (**A-i**) and delta area plot (**A-ii)** were presented. The overall survival (OS) of patients in each cluster was analyzed using KM (**B**). The expression of CXCL genes was analyzed between the clusters (**C**), and the immune components of the clusters were analyzed using the xCELL and TIMER methods (**D**, **E**). The immune checkpoints’ expression was also analyzed between the C3 and C4 clusters (**F**). The mutation condition of patients in the C3 and C4 clusters was also analyzed (**G**, **H**), as well as the correlation between VHL/RBPM1 mutation and the expression of CXCL genes (**I**).

**Figure 3:**
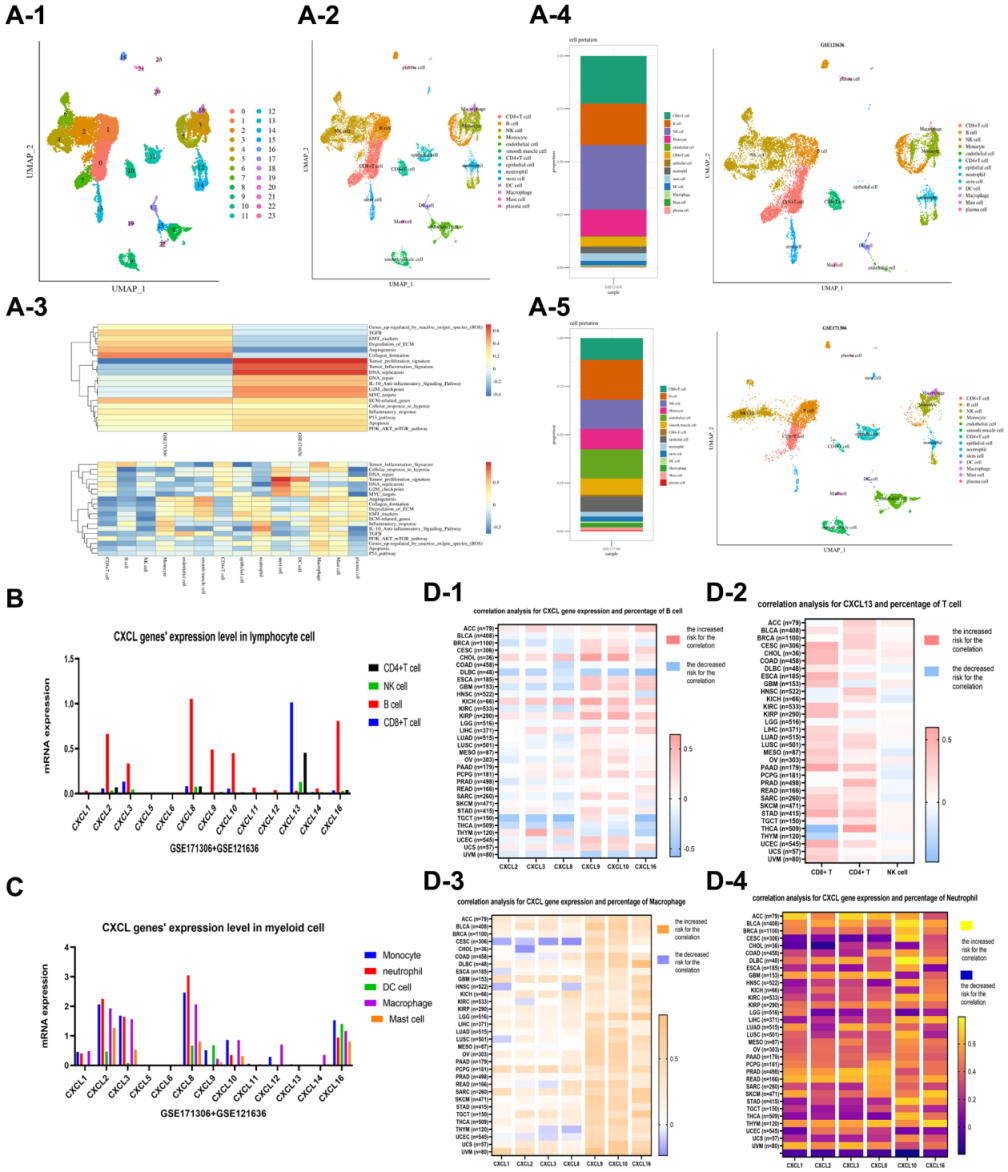
Single cell analysis was performed on two datasets, GSE121636 and GSE171306. 24 clusters were identified using the UMAP method, and 14 cell types were identified, including lymphocytes (CD8+ T cells, B cells, NK cells, and CD4+ T cells), myeloid cells (monocytes, macrophages, neutrophils, DC cells, and mast cells), and other cells (endothelial cells, epithelial cells, plasma cells, and stem cells) (**A-1** and **A-2**). An enrichment analysis for the cell components (**A-3**) was provided. The plots of cell components in the datasets of GSE121636 (**A-4**) and GSE171306 (**A-5**) were showed, respectively. The expression of CXCL genes was analyzed in lymphocyte and myeloid cells (**B**, **C**), and the correlation between CXCL genes and cell components was analyzed in a pan-cancer dataset, including CXCL genes and B cell (**D-1**), CXCL13 and T cell (**D-2**), CXCL genes and Macrophage (**D-3**), and CXCL genes and Neutrophil (**D-4**).

**Figure 4:**
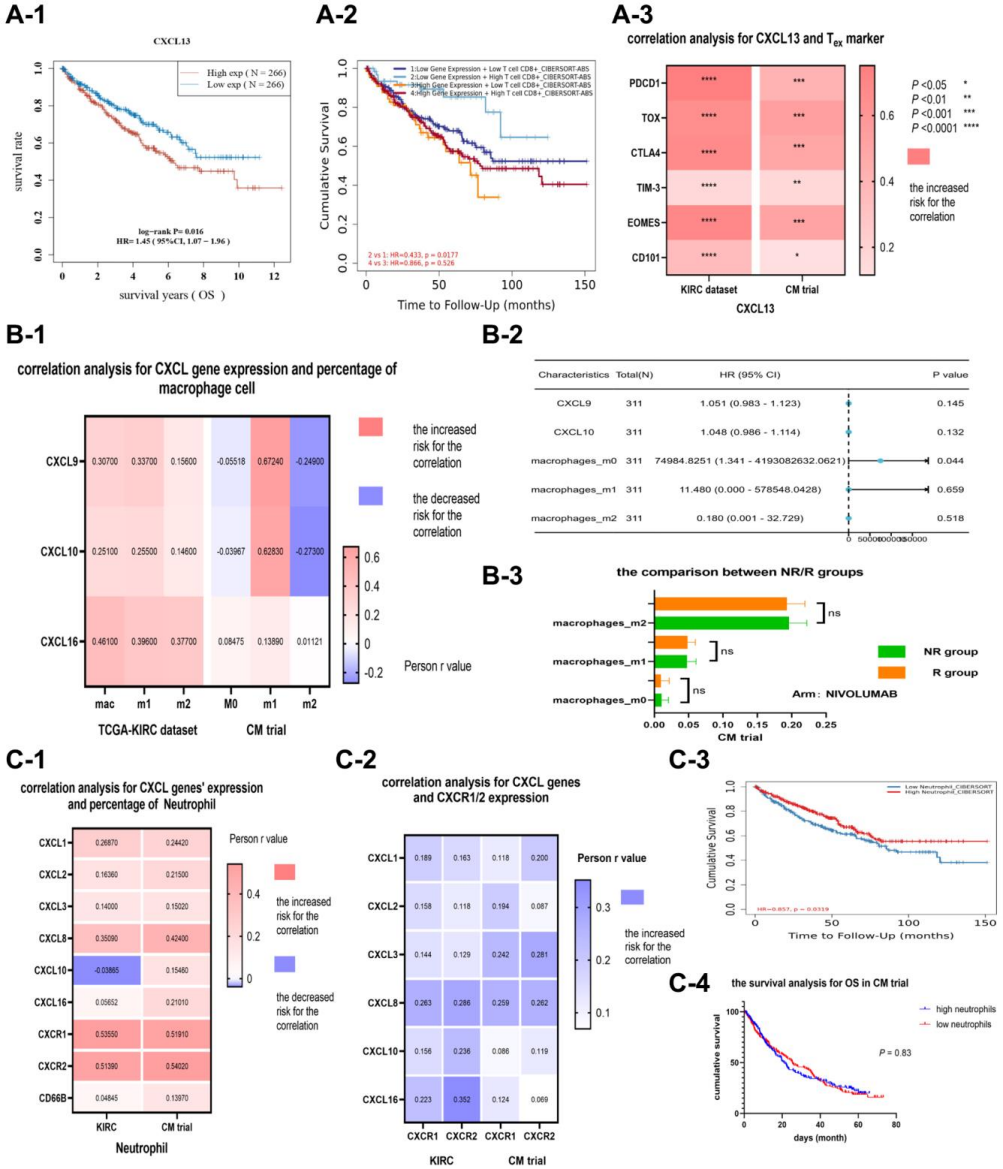
The correlation and prognostic analysis of CXCL13 and CD8 exhausted cells (**A**). The KM analysis (**A-1**) of CXCL13 in the KIRC dataset, (**A-2**) of CXCL13 and CD8+ T cell percentage combined. The correlation between CXCL13 and markers of CD8 exhausted cell (**A-3**). The correlation and prognostic value of CXCL9/10 and macrophage M1 cells (**B**). The correlation between CXCL9/10 and three types of macrophage cells (**B-1**), and the forest plot (**B-2**) showing CXCL9/10 expression level and the percentages of three types of macrophage cells in the KIRC dataset. The variance analysis (**B-3**) of three types of macrophage cells between immunotherapy response and non-response groups. The correlation and prognostic value of CXCL genes and neutrophil (**C**). The correlations (**C-1**) among CXCL genes, CXCR1/2, and the percentage of neutrophil in two datasets, as well as the correlation (**C-2**) between CXCL genes and CXCR1/2. The KM analysis (**C-3**) for the percentage of neutrophil in the KIRC dataset (*P* = 0.03), and the KM analysis (**C-4**) for the percentage of neutrophil in the CM dataset (*P* = 0.83).

Fourteen CXCL genes were found in the Gene Bank database (https://www.ncbi.nlm.nih.gov/gene/), including CXCL 1, 2, 3, 5, 6, 8, 9, 10, 11, 12, 13, 14, 16, and 17. The mRNA expression levels of these genes were then compared across three renal cancer datasets. 1. The mRNA expression of eight genes (CXCL 2, 5, 9, 10, 11, 13, 14, and 16) was increased in cancer tissue of the TCGA-KIRC dataset (Figure 1A-1) (*P* < 0.05), while only CXCL 12 was downregulated (*P* < 0.05). 2. The mRNA expression of eight genes (CXCL 1, 2, 5, 6, 8, 9, 10, and 11) was increased in cancer tissue of the GSE15641 dataset (Figure 1A-2) (*P* < 0.05). 3. The mRNA expression of nine genes (CXCL 2, 5, 6, 8, 9, 10, 11, 13, and 16) was increased in cancer tissue of the GSE14762 dataset (Figure 1A-3) (*P* < 0.05), while CXCL 12 and 14 were downregulated (*P* < 0.05). Further analysis showed that five CXCL genes (CXCL 2, 5, 9, 10, and 11) had increased mRNA expression in renal cancer tissue across all three datasets (Figure 1A-4).

### Prognostic value of CXCL expression in the TCGA-KIRC dataset

The prognostic significance of 14 CXCL genes in ccRcc was assessed based on the KIRC dataset. Seven CXCL genes (CXCL 1, 2, 3, 5, 8, 13, and 14) significantly impacted the overall survival (OS) (*P* < 0.05, Figure 1B-1–3), while CXCL 1, 5, and 13 significantly affected progression-free survival (FPS) (*P* < 0.05, Figure 1C-1–3). The data were represented based on forest plots and KM analysis.

### The mechanisms of CXCL gene expression in renal cancer: insights from unsupervised cluster analysis and immune component analysis

The unsupervised cluster analysis based on the KIRC dataset showed that CXCL gene expression levels significantly impacted the survival outcomes of renal cancer patients (Figure 2A). Moreover, the unsupervised cluster analysis identified four CXCL subtypes (Supplementary Table 2) with significantly different mRNA expressions (*P* < 0.05, Figure 2C). Further analysis using the KM method showed that the four subtypes had significantly different OS (Figure 2B), with subtype 3 having the worst OS and subtype 4 having the best OS (C1 vs. C3: *P* < 0.001; C2 vs. C3: *P* < 0.01; C4 vs. C3: *P* < 0.05).

The difference could be because of the differential expression of immune components. xCELL analysis showed that the immune components among the four subtypes (Figure 2D), including tumor suppressors (CD8+ effector, CD8+ central memory, Macrophage M1) and tumor promoters (CD4+ Th2, Macrophage M2), were significantly different (*P* < 0.001).

Further analysis revealed that the expression of six main immune cells (LAG3, SIGLEC15, CTLA4, HAVCR2, PDCD1LG2, and PDCD1) analyzed via TIMER were significantly different between subtypes C3 and C4 (*P* < 0.01, Figure 2E). For example, the expression of the cells was higher in subtype C3 than in subtype C4 (*P* < 0.001, Figure 2F).

The potential impact of VHL and PRBM1 mutations on the expression of CXCL genes and their tumor suppression function were also investigated. CXCL14 had the highest mutation rate (6%) in KIRC patients (Supplementary Figure 1A), while the other 13 CXCL genes had a mutation rate of less than 1%. The mutation rates of VHL (50%) and PRBM1 (49%) were higher in subtype C3 (Figure 2G) than in subtype C4 (less than 13%, Figure 2H). Finally, a correlation analysis indicated that the mutation of VHL and PRBM1 can influence the expression of CXCL genes (Figure 2I).

### Single cell analysis and pan-cancer analysis

**Figure 5:**
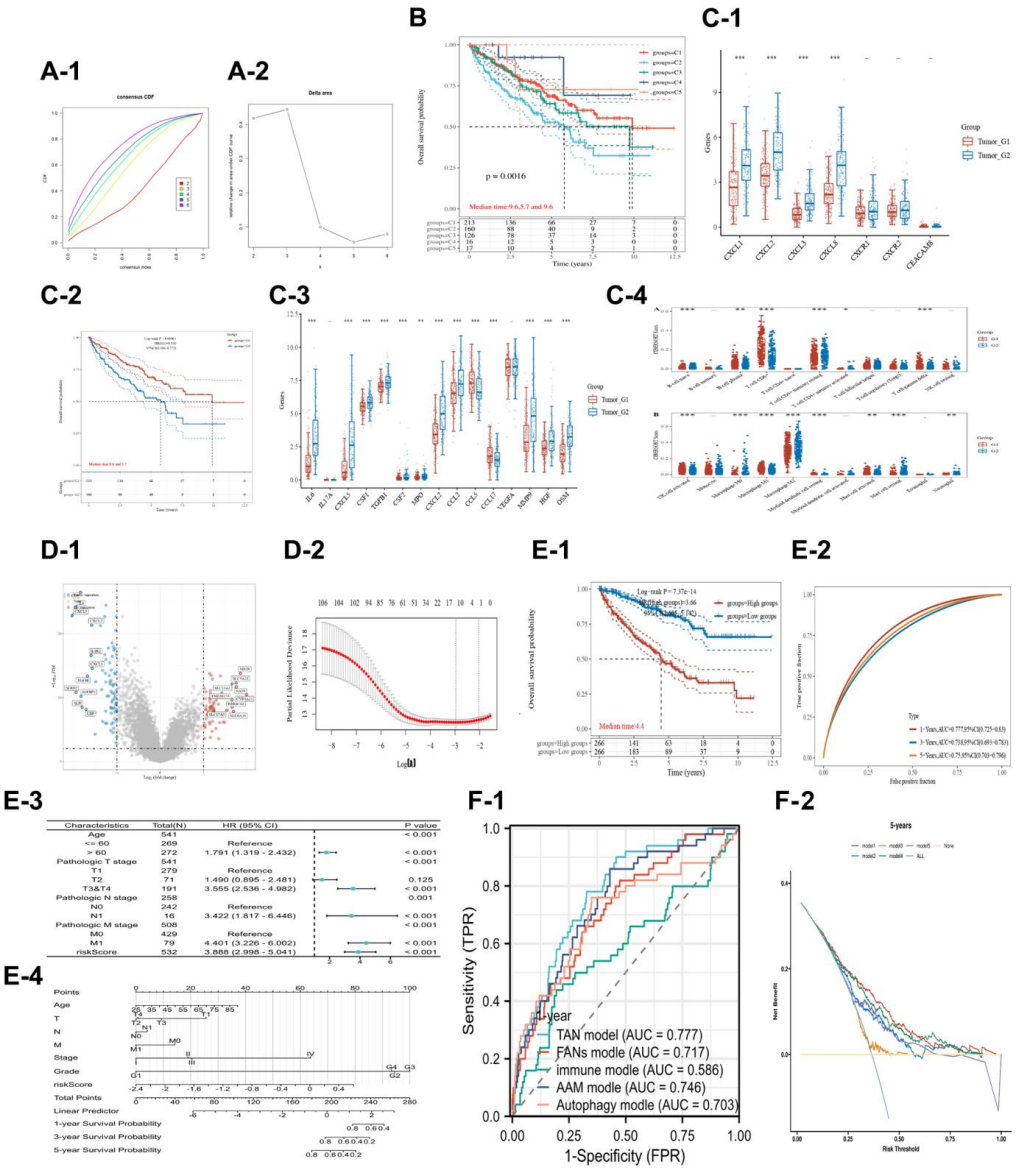
**Unsupervised cluster analysis for TAN 1/2 markers in the KIRC dataset.** The CDF plot (**A-1**) and delta area plot (**A-2**). (**B**) Indicates the KM analysis for the 5 subtypes. (**C**) Shows the prognostic value and TAN 2 markers for the subtype 1/2. The CXCL genes (**C-1**) for the subtype 1/2, the KM analysis (**C-2**) for subtype 1/2, the TAN 2 markers (**C-3**) between subtype 1/2, and the percentage of immune components (**C-4**) for the subtype 1/2. (**D**) Illustrates the different expression genes and lasso analysis for subtype 1/2. The volcano plot (**D-1**) and the process of lasso analysis (**D-2**). (**E**) Shows the prognostic value of TAN-related risk score in the KIRC dataset. The KM analysis (**E-1**) for different risk scores, the ROC time-dependent analysis (**E-2**), The forest plot of the univariate COX analysis for risk score and other clinical information (**E-3**), The nomogram (**E-4**). (**F**) Shows the comparison between 5 different renal cancer-related signatures. The ROC time-dependent analysis (**F-1**) and DCA analysis (**F-2**).

The function and mechanism of CXCL genes were evaluated using single-cell analysis based on two single-cell datasets of renal cancer, GSE171306 and GSE121636. Two samples of GSE171306 (GSM5222644 and GSM5222645) and three samples of GSE121636 (GSM3440844, GSM3440845, and GSM3440846) underwent detailed analysis after standard data preprocessing. The UMAP method identified 24 distinct cell clusters based on 27049 cells (Figure 3A-1). The top ten genes with the highest expression in each cluster were used to assign cell lineages, resulting in the identification of 14 cell types (Figure 3A-2), including lymphocytes (CD8+ T cells, B cells, NK cells, and CD4+ T cells), myeloid cells (monocytes, macrophages, neutrophils, DC cells, and mast cells), and other cellular units (endothelial cellular units, epithelial cellular units, plasma cellular units, and stem cellular units). Two separate plots were drawn for GSE121636 (Figure 3A-3) and GSE171306 (Figure 3A-5). A heat plot of enrichment analysis was also drawn (Figure 3A-4).

The expression and distribution patterns of the CXCL genes were analyzed via single-cell analysis (Figure 3B, 3C). CXCL 13 was upregulated in CD8+ T cells and CD4+ T cells, while CXCL 2, CXCL 3, CXCL 8, CXCL 9, CXCL 10, and CXCL 16 were upregulated in B cells. However, CXCL genes were downregulated in NK cells. The interaction between CXCL genes and myeloid cells showed that CXCL 2, CXCL 3, CXCL 8, and CXCL 16 were upregulated in multi-myeloid cells, such as monocytes, macrophages, and neutrophils, while CXCL 1, CXCL 9, and CXCL 10 were only upregulated in macrophages and neutrophils.

The interaction between CXCL genes and immune components in a pan-cancer dimension was also evaluated. The expressions of CXCL genes in 33 types of TCGA datasets were estimated. The immune components of 33 types of TCGA cancer patients were evaluated using the CIBERSORT method. The correlation between CXCL 2, CXCL 3, CXCL 8, CXCL 9, CXCL 10, and CXCL 16 expressions and B cell percentage in different cancers was unclear (Figure 3D-1). Furthermore, the expression of CXCL 13 was positively correlated with CD8+ T cell percentage in most cancers, while the correlation between CXCL13 expression with CD4+ T cell and NK cell was not identified (Figure 3D-2). The expression of CXCL 9 and CXCL 10 was positively correlated with macrophage percentages in most cancers, especially with macrophage M1 (Figure 3D-3). Also, the expressions of CXCL 1, CXCL 2, CXCL 3, CXCL 8, CXCL 10, and CXCL 16 positively influenced the percentages of neutrophils in most cancers (Figure 3D-4).

The interaction among CXCL genes was analyzed based on the KIRC dataset. The results showed that the expression of two clusters (clusters 1 and 2) was positively correlated. Cluster one included CXCL 1, CXCL 2, CXCL 3, CXCL 5, CXCL 6, and CXCL 8 (correlation *R*-value = 0.22 ~ 0.76, Supplementary Figure 1B), and cluster two included CXCL 9, CXCL 10, and CXCL 13 (correlation *R*-value = 0.59 ~ 0.95).

### The relationship between CXCL 13 with CD8+-exhausted T cells, CXCL 9/10, macrophage M1 cells, CXCL 1/2/3/8, and neutrophils in renal cancer patients

#### CXCL 13 and exhausted CD8 T cells

CXCL13 expression was positively correlated with CD8+ T cell percentage in most cancer types. Furthermore, CXCL13 expression was significantly upregulated in CD8+ T cells. However, KM analysis revealed that high CXCL 13 levels in renal cancer patients resulted in worse survival outcomes (Figure 4A-1), while high CD8+ T levels resulted in better survival outcomes (Figure 4A-2). The relationship between CXCL13 expression and the divergent prognostic outcomes of CD8+ T cells in renal cancer patients was further evaluated via correlation analysis. Several markers of CD8+-exhausted T cells, including PDCD1, TOX, CTLA4, TIM-3, EOMES, and CD101, in both the KIRC and CM datasets, were analyzed. Results revealed that CXCL13 expression was related to CD8-exhausted T cell markers in both datasets (*P* < 0.05), especially between CXCL13 expression and four markers (PDCD1, TOX, CTLA4, and EOMES) (*P* < 0.001, Figure 4A-3). These findings suggest that CXCL13 may serve as a novel marker for CD8+-exhausted T cells. A high CXCL13 expression in renal cancer patients may indicate the presence of several CD8+-exhausted T cells, suggesting that it can suppress immune function.

#### CXCL 9/10 and macrophage M1 cells

The expression of CXCL 9/10 was significantly associated with M1 cell percentage in most cancer types, especially in the KIRC and CM datasets, (compared with M2 cells) (Figure 4B-1). However, the correlation between CXC L9/10 and M1 cells had no direct advantage for renal clear cell carcinoma patients (Figure 4B-2, 4B-3).

#### CXCL 1/2/3/8 and neutrophil

CXCR1/2 can recruit neutrophils from the blood vessel into the tumor. Besides, CXCL-CXCR is a common molecular pairing model. In this study, CXCR 1/2 expression was significantly correlated with neutrophil percentage (Figure 4C-1). Moreover, CXCL 8 expression was significantly associated with CXCR 1/2 expression (Figure 4C-2). The prognostic value of neutrophil percentage was explored using KIRC and CM datasets. Furthermore, Kaplan-Meier analysis showed that neutrophil percentage can predict OS in the KIRC cohort (*P* = 0.03, Figure 4C-3). However, the correlation between neutrophil percentage with OS or PFS in the CM dataset was not significantly different (*P* > 0.05, Figure 4C-4).

Although neutrophil density in the tumor was not a significant prognostic index, neutrophil polarization may be an important prognostic index.

### Neutrophil polarization is an important prognostic factor for renal cancer patients

Neutrophil polarization is crucial in survival outcomes. Different neutrophil phenotypes are associated with different survival outcomes. For instance, type 1 neutrophils (TAN 1) can promote inflammation and suppress tumor growth, while type 2 neutrophils (TAN 2) promote tumor growth.

In this study, TAN 1/2 markers were extracted from relevant studies to assess the prognostic value of neutrophil polarization in patients with clear cell renal cancer. The patients in the KIRC dataset were subjected to an unsupervised cluster analysis (Figure 5A-1, 5A-2). Five subtypes had significant prognostic value and were used for further analysis (Supplementary Table 3) (Figure 5B). The expression analysis of CXCL and CXCR 1/2 (Figure 5C-1), survival analysis (Figure 5C-2), immune component analysis (Figure 5C-4), and comparison of TAN 1/2 marker expression (Figure 5C-3) were then performed to further analyze the five subtypes. The C1/2 subtypes were classified as TAN 1/2 phenotypes (213 patients in the C1 subtype and 160 patients in the C2 subtype). Compared with the C2 subtype, the C1 subtype exhibited a more active immune function, characterized by huge percentage of activated CD8 T cells, NK cells, M1 macrophages, and mast cells. Meanwhile, the C2 subtype promoted tumor progression and immune suppression and was characterized by high percentages of resting CD4+ T cell memory, M2 macrophages, resting DC cells, resting mast cells, and neutrophils. Further comparison analysis showed that most TAN 2 markers were highly expressed in the C2 subtype (*P* < 0.05). Furthermore, KM analysis indicated that the C2 subtype was associated with worse survival outcomes (OS, *P* = 0.0061; FPS, *P* < 0.0001). The expressions of CXCL 1/2/3/8 and CXCR 1/2 were higher in the C2 subtype than in the C1 subtype (*P* < 0.001). However, CXCR 1/2 expression was not significantly different between the two subtypes (*P* > 0.05).

Moreover, a neutrophil polarization signature and related risk score were constructed. The neutrophil-related signature (TAN signature) was developed in three steps as follows: 1. Differentially expressed genes (DEGs) between the two subtypes were identified via the “limma” package in R (*P* < 0.01, Figure 5D-1 and Supplementary Table 4); 2. The DEGs with prognostic value (*P* < 0.01) were further selected in the KIRC cohort based on OS analysis (Supplementary Table 5); [3]. Lasso regression analysis was conducted, and the TAN signature was constructed using the remaining genes (Figure 5D-2). A total of 150 DEGs were identified (69 upregulated in the C1 subtype, 81 upregulated in the C2 subtype), of which 107 genes exhibited significant survival values in the KIRC dataset. Finally, the TAN signature was constructed using 11 genes (BBOX1, TMEM125, SLC16A12, LRRC19, SAA1, SLC38A5, SMIM24, PLAUR, CXCL1, CXCL2, and CXCL5). Functional enrichment analysis indicated that the DEGs were significantly involved in cytokine and cytokine receptor functions, and cytokine and cytokine receptor interactions in the C2 subtype. Further analysis also revealed that CXCL 2/3/5 were among the significant DEGs.

The TAN risk score was developed using signature and LASSO regression techniques as follows:

Risk Score = (−0.0159) × BBOX1 + (−0.0007) × TMEM125 + (−0.146) × SLC16A12 + (−0.043) × LRRC19 + (0.0217) × SAA1 + (0.0063) × SLC38A5 + (−0.0701) × SMIM24 + (0.0179) × PLAUR + (0.0332) × CXCL1 + (0.0141) × CXCL2 + (0.0061) × CXCL5.

KM analysis (Figure 5E-1), time-dependent ROC analysis (Figure 5E-2), and COX regression analysis (Figure 5E-3) were performed to assess the prognostic value of the TAN risk score in patients with renal cancer. The analyses confirmed that the TAN risk score had some prognostic value in KIRC patients (*P* < 0.001) in the KM analysis and COX regression analysis: AUC = 0.77, 0.73, 0.75 after one, = 0.73, after three, and = five years, respectively (ROC analysis). The nomogram map (Figure 5E-4) showed that the risk score was significantly associated with clinical features, such as age and TNM stages. Compared with four other renal cancer prognostic signatures, ROC analysis (Figure 5F-1) and DCA (Figure 5F-2) showed that the TAN signature had the largest AUC value and the maximum area under the curve, respectively.

These results indicate neutrophil polarization is a significant prognostic factor for patients with renal clear cell cancer. CXCL 1/2/3/8 were upregulated in the TAN 2 subtype and thus may be positively correlated with the TAN 2 phenotype. CXCL 8/CXCR1-2 promotes cancer progression in various cancers, while CXCL 2/CXCR1 facilitates renal clear cell carcinoma.

### CXCL2-CXCR1 axis is crucial for neutrophil recruitment and polarization

**Figure 6:**
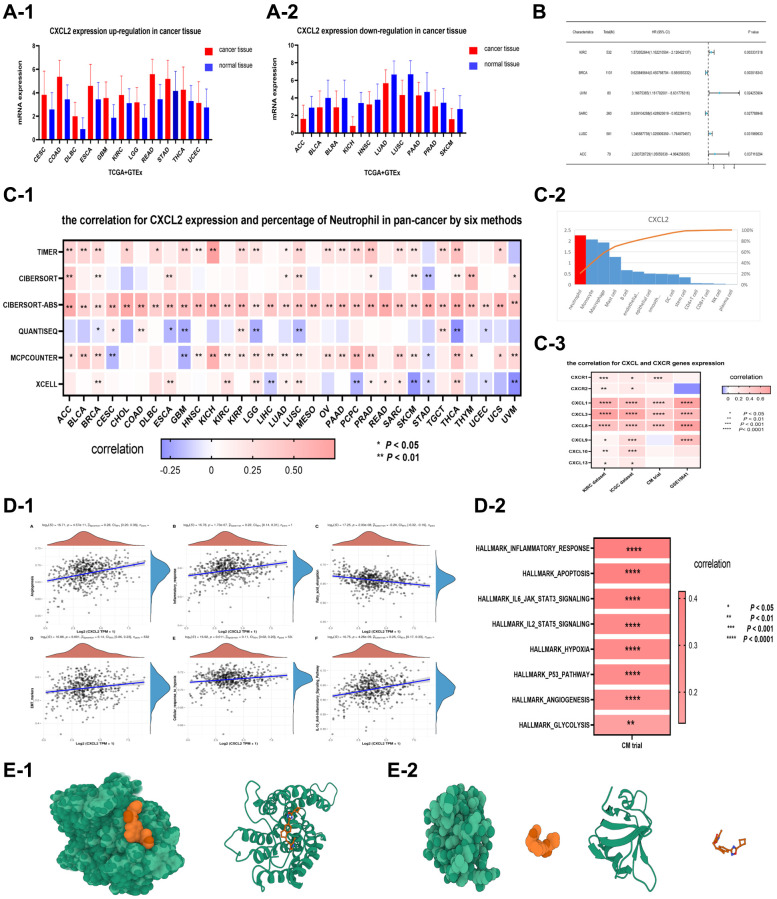
CXCL2 expression was upregulated in 11 types of cancer (CESC, COAD, DLBC, ESCA, GBM, KIRC, LGG, READ, STAD, THCA, UCEC), with *P* < 0.05 (**A-1**). On the other hand, CXCL2 expression was significantly (*P* < 0.05) downregulated in 10 types of cancer (ACC, BLCA, BLRA, KICH, HNSC, LUAD, LUSC, PAAD, PRAD, SKCM) (**A-2**). (**B**) Shows that CXCL2 was significantly (*P* < 0.05) associated with the survival value of 6 types of cancers (KIRC, BRCA, UVM, SARC, LUSC, ACC). The relationship between CXCL2 expression and neutrophils in various types of cancer. The correlation between CXCL2 expression and the percentage of neutrophils in pan-cancer dataset as determined by 6 different methods (**C-1**). CXCL2 expression in different types of cells. CXCL2 expression was highest in neutrophils (**C-2**). The correlation between CXCL2 expression and other CXCL genes, specifically CXCR1 and CXCR2 (**C-3**). The expression level of CXCL2 was positively correlated with various pro-tumor pathways in two renal cancer datasets. In the first dataset of KIRC (**D-1**), CXCL2 expression level was positively correlated with 6 pro-tumor pathways, including Angiogenesis, Inflammatory response, fatty acid elongation, EMT pathway, cellular response to hypoxia, and anti-inflammatory signaling pathway (*P* < 0.01). In the second dataset (**D-2**), CXCL2 expression was positively correlated with 9 key pro-tumor pathways (*P* < 0.01), such as apoptosis, hypoxia, P53 pathway, and glycolysis. Molecular docking analysis of CXCL2 and its receptors. Interaction models between CXCL2 and CXCR1 (**E-1**) and CXCR2 (**E-2**) were developed. These models provided insights into the molecular mechanisms behind the positive correlations between CXCL2 expression and pro-tumor pathways.

The expression levels of CXCL2 varied among various cancers. For example, CXCL 2 was upregulated in 11 cancers (Figure 6A-1) and downregulated in 10 cancers (*P* < 0.05, Figure 6A-2). CXCL2 also had survival value in six cancers (*P* < 0.05, Figure 6B), especially in renal clear cell carcinoma (*P* = 0.003).

CXCL2 is crucial in neutrophil recruitment into the tumor tissue. Herein, CXCL2 expression was correlated with neutrophil percentage across multiple cancers based on six immune analysis methods (Figure 6C-1), suggesting that neutrophil percentage increases with increasing CXCL2 expression level. Furthermore, CXCL 2 expression was significantly associated with CXCL 1/3/8 expression in four datasets of renal clear cell carcinoma, especially CXCR 1 (Figure 6C-3). CXCR 1/2 are crucial in recruiting neutrophils from blood vessels, while CXCL 1/3/8 activates CXCR 1/2 function. These findings suggest that CXCL 2 stimulates CXCR 1 and thus can recruit neutrophils into the cancer tissue. Also, single-cell analysis revealed that CXCL 2 was significantly upregulated in neutrophils (Figure 6C-2), indicating that the neutrophils recruited by CXCL 2 can secrete more CXCL 2 and recruit additional neutrophils.

CXCL 2 participates in neutrophil polarization towards TAN 2 phenotype within tumor tissue. In this study, CXCL2 expression was upregulated in TAN 2 phenotype (C2 subtype). Besides, CXCL2 expression was positively correlated with TAN risk score (*P* < 0.0001, Supplementary Figure 1E). GSVA analysis revealed that CXCL2 expression was associated with many pathways, such as apoptosis, angiogenesis, IL-10 anti-inflammatory signaling, cellular response to hypoxia, and glycolysis gluconeogenesis based on the KIRC dataset (*P* < 0.01, Figure 6D-1). CXCL2 expression was associated with many pathways with the pathways in the CM dataset, such as apoptosis, angiogenesis, hypoxia, and glycolysis (*P* < 0.01, Figure 6D-2).

Further analysis showed that the CXCL2-CXCR1 axis is a potential molecular docking site. Although CXCR 1/2 are potential receptors for CXCL2, CXCL2-CXCR1 is more likely a molecular docking site because CXCL 2 expression was significantly correlated with CXCR1 in three renal cancer datasets (KIRC, *P* < 0.001; ICGC, *P* < 0.05; CM, *P* < 0.001), not significantly correlated with CXCR2 in the KIRC and ICGC datasets. Besides, CXCL 2 expression was negatively correlated with CXCR 2 in the GSE15461 dataset. The potential molecular docking between CXCL 2 and CXCR 1/2 was further analyzed using AutodockVina. Five potential binding sites for CXCL2-CXCR1 with low binding energies (−48.98, −48.2, −48.15, −47.96, and −47.63 kcal/mol, respectively) were detected, indicating high stability (Figure 6E-1 and Supplementary Figure 1C). Moreover, one potential binding site with a high binding energy of 4759 kcal/mol was also identified, indicating low stability (Figure 6E-2 and Supplementary Figure 1D).

Therefore, these results indicate that CXCL2 activates CXCR 1 and recruits neutrophils into the tumor tissue through interaction with CXCR 1. Additionally, CXCL2 promotes neutrophil polarization within the tumor towards the TAN 2 phenotype, thus facilitating cancer progression. Furthermore, CXCL2 can promote the growth, penetration, and movement of renal clear cell carcinoma cells in a laboratory setting through the activation of the EMT process.

### The CXCL2 enhanced the proliferation, invasion and migration of renal clear cell carcinoma’s cells by activating the EMT pathway

**Figure 7:**
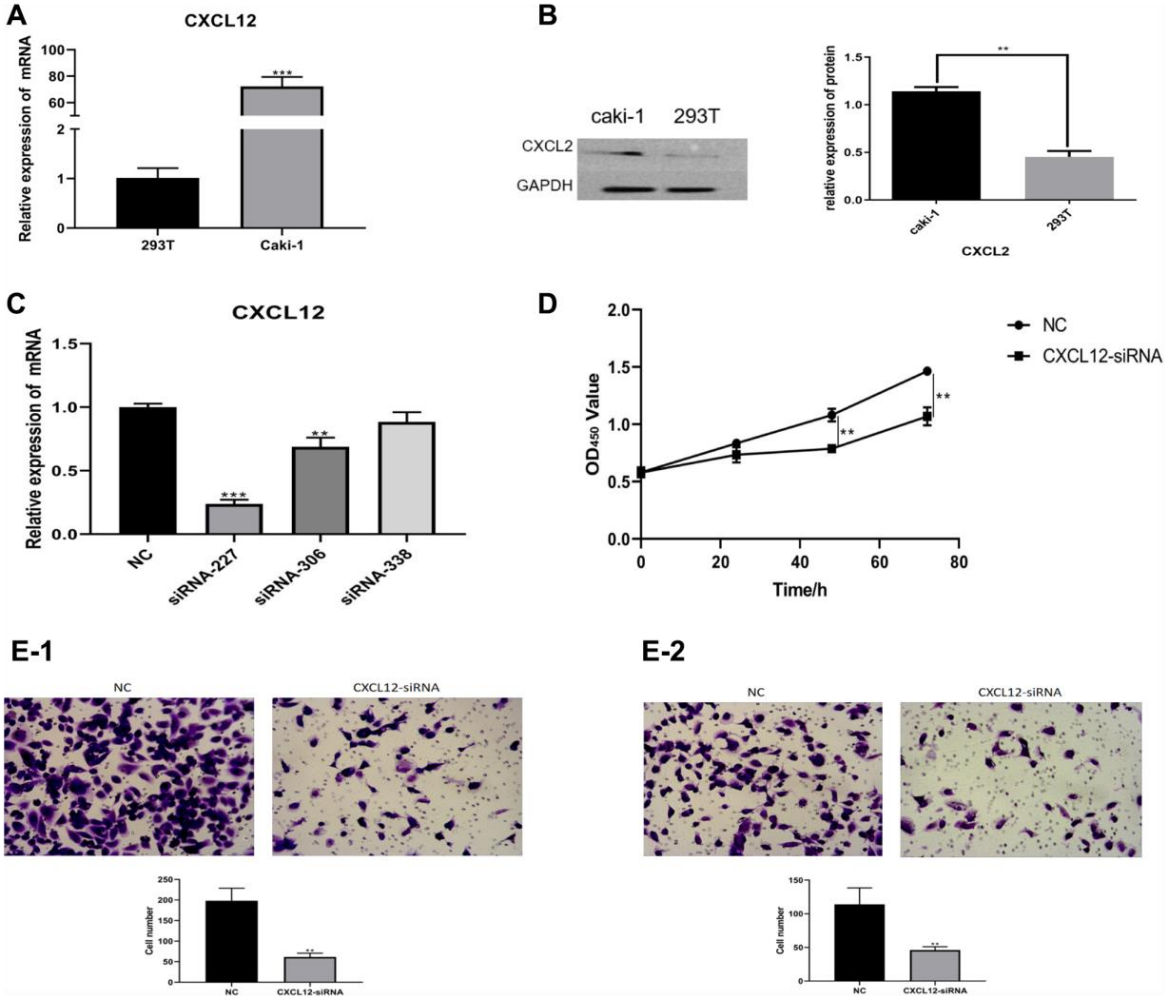
The mRNA expression of CXCL2 was measured using Q-PCR (**A**). The protein expression of CXCL2 was measured using western blotting (**B**). In the caki-1 cell line, the down-regulation of CXCL2 was performed (**C**). The impact of CXCL2 down-regulation on cell proliferation was measured using CCK-8 assay (**D**). The impact on migration (**E-1**) and invasion (**E-2**) was measured using Transwell assay.

The expression of CXCL2 at mRNA and protein levels was measured by Quantitative PCR (Q-PCR) and western blotting. The results showed that the mRNA and protein levels of CXCL2 in the caki-1 cell line were higher (*P* < 0.01, Figure 7A, 7B) compared with levels in the 293T cell line. To explore the correlation between CXCL2 expression and the function of renal clear cell carcinoma cells, CXCL2 was downregulated in the caki-1 cell line. Three caki-1 cell lines with CXCL2 knockdown were generated, and the cell line with the lowest CXCL2 expression (siRNA-227 cell line) was selected for further functional experiments (Figure 7C).

Functional comparison between the caki-1 cell line and siRNA-227 cell line was performed in terms of proliferation, invasion, and migration. The results showed that the siRNA cell line exhibited a weaker proliferation ability (determined by CCK-8 assay, *P* < 0.01, Figure 7D), reduced invasion (Transwell assay, *P* < 0.01, Figure 7E-1), and migration (Transwell assay, *P* < 0.01, Figure 7E-2) compared with the caki-1 cell line.

**Figure 8:**
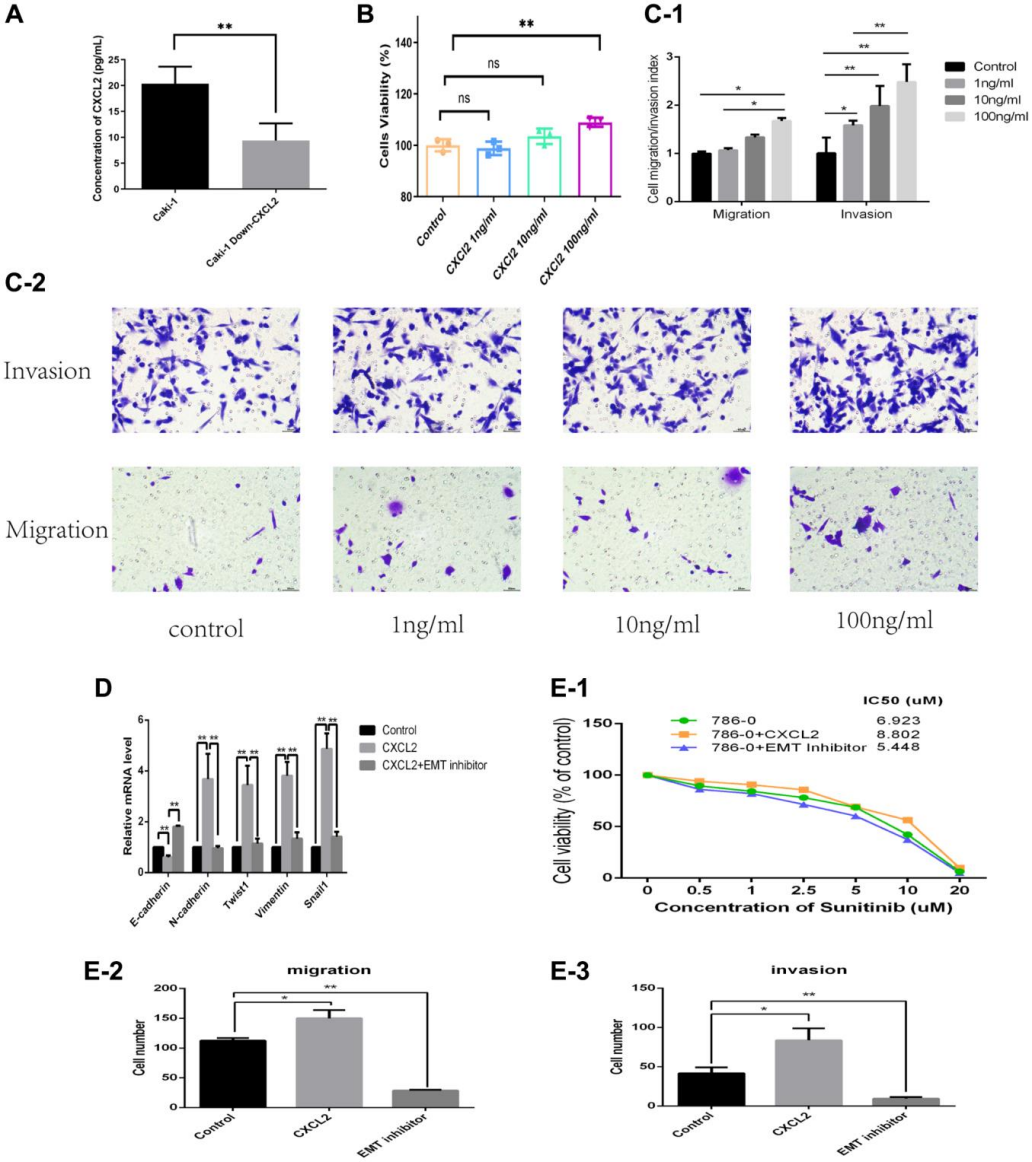
The protein concentration of CXCL2 in the extracellular medium as determined using ELISA (**A**). The impact of three concentrations of human recombinant CXCL2 protein on cell proliferation as determined by the CCK-8 assay (**B**). The impact on migration and invasion as evaluated using the Transwell assay (**C-1**, **C-2**). The mRNA expression of EMT markers was in three different cell culture environments (**D**). The migration, invasion, and resistance to sunitinib outcomes in three different cell culture environments (**E-1**–**E-3**).

The protein concentration of CXCL2 in the extracellular medium of renal clear cell carcinoma has been identified as an important factor contributing to cancer development. The protein concentration of CXCL2 in the medium was first compared between the siRNA-227 cell line and caki-1 cell line. Analysis of ELSA results demonstrated that CXCL2 was downregulated in the siRNA-227 cell line (*P* < 0.01, Figure 8A). Furthermore, the effects of human recombinant CXCL2 protein (SB, product ID: 10586-HNCE) on the cell proliferation, infiltration, and migration were investigated by incubation with three different concentrations of the caki-1 cell culture medium (1 ng/ml, 10 ng/ml, and 100 ng/ml). The results indicated that the caki-1 cell line which had the highest concentration of human recombinant CXCL2 protein (100 ng/ml, CXCL2 group) exhibited increased proliferation activity (*P* < 0.01, Figure 8B), increased invasion (*P* < 0.01, Figure 8C-1, 8C-2), and migration (*P* < 0.01, Figure 8C-1, 8C-2) compared with the control group (NC). Furthermore, the caki-1 cell line with 100 ng/ml human recombinant CXCL2 protein showed increased resistance to sunitinib (MCE, product ID: SU11248) compared with the NC group (NC group vs. CXCL2 group IC50: 6.9 vs. 8.8 μm, Figure 8E-1).

Considering that CXCL2 promoted the progression of renal clear cell carcinoma via inducing activation of the EMT process, we added the EMT pathway inhibitor (EMT inhibitor-1, 10 uM/mL, product ID: HY-101275) to the medium containing 100 ng/mL of human recombination CXCL2 protein (EMT group). Subsequently, invasion, migration, and sunitinib resistance were then explored in three cell lines (CXCL2 group, EMT group, and NC group). Results indicated that the EMT group had reduced invasion and migration abilities (Figure 8E-2, 8E-3 and Supplementary Figure 2) compared with the CXCL2 group. Moreover, the IC50 test revealed that the EMT group had a lower value relative to the CXCL2 group (5.4 vs. 8.8 uM, Figure 8E-1). The expression of markers of the EMT pathway (E-cadherin, N-cadherin, Twist1, Vimentin, and Snail1) in the three groups was quantified by Q-PCR. Results showed that while E-cadherin was lower in the CXCL2 group, N-cadherin, Twist1, Vimentin, and Snail1 were higher compared with levels in the NC group. Further analysis showed that E-cadherin was increased while N-cadherin, Twist1, Vimentin, and Snail1 were decreased in the EMT group (Figure 8D) compared with levels in the CXCL2 group. These findings suggest that addition of the human recombination CXCL2 protein activated the EMT pathway in the caki-1 cell line, and activated CXCL2 inhibited the EMT pathway.

## DISCUSSION

The function of CXCL genes, particularly CXCL8, in tumor development has been extensively studied [19]. CXCL8, the key ligand of CXCR1/2, is overexpressed in various solid tumors and has been shown to promote tumor development [20]. However, the functions of other CXCL genes in different types of cancers are largely unknown. This study highlights the crucial role of CXCL genes in patients with ccRCC. Specifically, the expression level of the genes was associated with the survival outcomes of patients. A single-cell analysis revealed three pairs of CXCL genes and immune cells, which were validated through pan-cancer analysis. The present results demonstrate that CXCL genes can influence cancer progression by regulating immune cells.

In particular, our results showed that elevated expression of CXCL13 was correlated with poor survival outcomes in ccRCC patients. Moreover, we found a positive correlation between the expression levels of CXCL13 and CD8+ exhausted T cells, which is in line with findings from previous reports in other types of solid tumors. The relationship between elevated levels of CXCL13 and negative outcomes in patients has been previously reported in several solid tumors [21]. The CXCL13-CXCR5 axis, which drives B cell recruitment, is believed to have a vital role in this phenomenon [22]. In a 2021 study, high expression of CXCL13 was correlated with high levels of CD8+ T cells in ovarian cancer, and the authors demonstrated that the CXCL13-CXCR5 axis was involved in CD8+ T cell recruitment [23]. A single-cell meta-analysis of nine datasets from immunotherapy (ICI) trials revealed that CD8+ T cells expressing CXCL13 can be potential predictors of ICI outcomes, with CXCL13 being recognized as a marker for CD8+ exhausted cells in 2022 [24]. In our study, the conflicting prognostic results for CXCL13 and CD8+ T cells in ccRcc patients could not be explained initially. However, further analysis revealed a significant positive correlation between the expression levels of CXCL13 and markers for CD8+ exhausted T cells, which provided a hypothesis suggesting a potential link between CXCL13 and CD8+ exhausted T cells. Further investigations are required to fully explore the function of CXCL13 in this context.

In this study, single-cell and pan-cancer analysis revealed that CXCL9/10 plays a role in M1-polarized tumor-associated macrophages in ccRcc patients. M1 macrophages produce angiostatic substances which regulate the development of immunity response against cancer and CXCL10 has been identified as a marker of M1 macrophages. Furthermore, in a single-cell analysis of breast cancer [25, 26], it was found that high expression of CXCL9/10 in M1 macrophages was linked to better response to immune checkpoint inhibitor therapy. Similarly, high expression of CXCL9/10 was observed in macrophage cells in renal cancer datasets, with a strong correlation with M1 macrophages in the KIRC and CM datasets. However, our analysis of CXCL9/10 levels and the percentage of M1 cells did not reveal any significant association with prognostic results or response to immune checkpoint inhibitors in ccRcc patients.

CXCL1/2/3/8, on the other hand, was significantly associated with the overall survival of ccRcc patients. In addition, there was a correlation between CXCL1/2/3/8 and neutrophils in renal clear cell carcinoma. To understand the potential mechanism of these genes and neutrophil in ccRcc patients, we analyzed the density of neutrophil in renal cancer. Activation of CXCR1/2 by CXCL genes is a key factor regulating the recruitment of neutrophils into the cancer site [27]. However, the percentage of neutrophils in ccRcc patients was not found to be an independent prognostic indicator, despite the strong association of CXCR1/2 with the density of neutrophil, as well as between CXCL1/2/3/8 and CXCR1/2 in the two ccRcc patient datasets. Another potential mechanism is the neutrophil polarization. In our analyses, we found that different TAN phenotypes had distinct influence on the prognostic outcomes. TAN1/2 phenotypes had pro-tumor or tumor suppressor properties in various types of cancers, such as melanoma [28], head and neck cancer [29], and HCC [30]. Several CXCL genes have been recognized to be potential markers of TAN2 phenotype, such as CXCL5, which has been reported to independently predict reduced overall survival duration and total risk of reoccurrence in liver cancer patients [31]. CXCL8 can stimulate the transformation of neutrophils towards the TAN2 phenotype by upregulating GM-CSF and HGF in HCC tissues [32]. In our study, we identified more than 100 genes with significant prognostic value between TAN1/2 phenotypes, suggesting that the mechanisms of neutrophil polarization may be driven by multiple genes. Additionally, we constructed a TAN phenotype-based signature and risk score for ccRcc patients. The performance of the signature was compared with four other renal cancer signatures. Results showed that our signature had the highest AUC value based on the ROC and maximum area under the curve in DCA, suggesting that TAN phenotype has a more significant function in ccRcc patients compared with the immune phenotype [33], fatty acid metabolism [34], amino acid metabolism [35], and autophagy [36].

Moreover, our results revealed that CXCL2 promoted renal clear cell carcinoma progression through multiple mechanisms. Firstly, CXCL2 (one ligand of CXCR1/2) was associated with tumor-associated neutrophil recruitment in the KIRC and CM datasets. Further analysis of molecular docking results confirmed the stable binding of CXCL2 to CXCR1. Secondly, CXCL2 may serve as a novel marker of TAN 2 phenotype, and is strongly associated with the TAN 2 phenotype functions such as apoptosis, angiogenesis, anti-inflammatory signaling, hypoxia, and glycolysis-gluconeogenesis. Thirdly, CXCL2 was found to directly enhance the malignant features of carcinoma cells such as proliferation, invasion, and migration. The EMT pathway, as analyzed through GSVA in KIRC, was verified to be a key mechanism by which CXCL2 regulated renal clear cell carcinoma cells.

Furthermore, we identified three pairs of interactions between CXCL genes and the specific immune cells in renal clear cell carcinoma samples: CXCL13-CD8+ exhausted T cells, CXCL 9/10-M1, and CXCL 1/2/3/8-neutrophil polarization for cancer. However, the mechanisms regulating these interactions are poorly understood, and thus further *in vivo* experiments are advocated to provide deeper understanding of the intricate interactions between TME and cancer cells. To further test the function of CXCL2 on the tumor microenvironment and neutrophil polarization, we plan to conduct additional *in vivo* experiments.

## CONCLUSION

Seven CXCL genes (CXCL 1/2/3/5/8/13/14) can influence the prognosis of ccRcc patients, and three pairs of interactions between CXCL genes and immune cells (CXCL13- CD8+ exhausted T cells, CXCL 9/10 and M1 cells, CXCL 1/2/3/8 and neutrophils) were identified in this study. CXCL2 can attract and regulate neutrophils thereby participate in the progression of ccRcc.

## Supplementary Materials

Supplementary Materials

Supplementary Figures

Supplementary Table 1

Supplementary Table 2

Supplementary Table 3

Supplementary Table 4

Supplementary Table 5
