# Supplementary material for: Prognostic significance and mechanisms of CXCL genes in clear cell renal cell carcinoma
Source: Aging (Albany NY). 2023 Aug 3;15(16):7974–96. doi: 10.18632/aging.204922 (PMC10497021; doi:10.18632/aging.204922)
Supplement: Supplementary Materials [file aging-15-204922-s001.pdf]

SUPPLEMENTARY MATERIALS

One year ago, we authored the initial draft of our research paper and submitted it to the pre-print section of Nature Research Square (https://www.researchsquare.com/article/rs-1550360/v1). Over the past year, we conducted a comprehensive series of bioinformatics studies and *in vitro* experiments, incorporating the novel findings into the revised version of our paper. Upon comparison of the current version with the initial draft, we discovered that Figure 7 was present in both iterations, whereas several other figures have been omitted.
