# Supplementary material for: Prognostic significance and mechanisms of CXCL genes in clear cell renal cell carcinoma
Source: Aging (Albany NY). 2023 Aug 3;15(16):7974–96. doi: 10.18632/aging.204922 (PMC10497021; doi:10.18632/aging.204922)
Supplement: Supplementary Figures [file aging-15-204922-s002.pdf]

SUPPLEMENTARY FIGURES

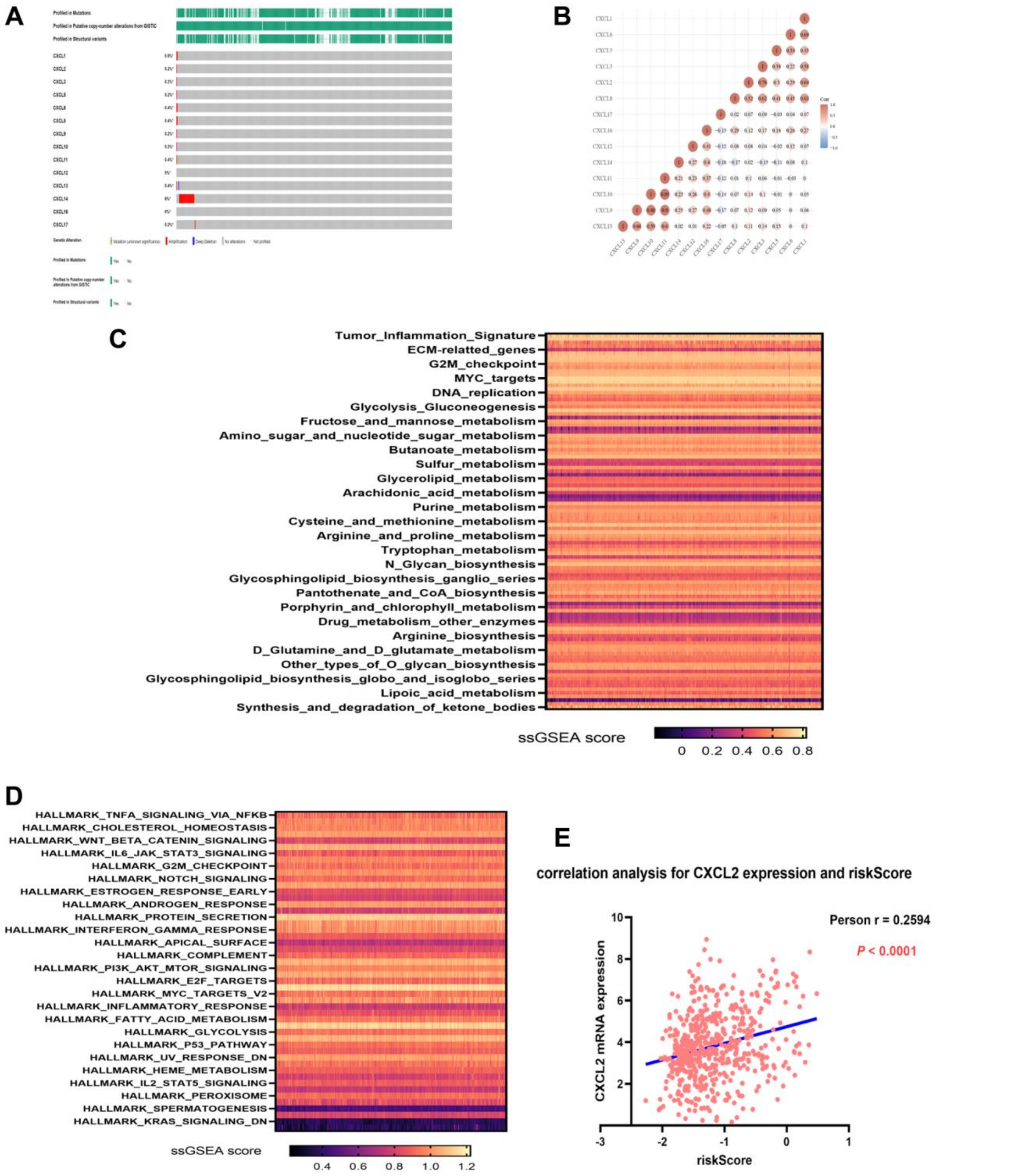

Supplementary Figure 1. (**A**) The mutation condition of CXCL genes in the KIRC dataset; (**B**) The correlation between CXCL genes in KIRC the dataset; (**C**, **D**) The correlation between CXCL2 gene expression and pathway ssGSVA score, c KIRC, d CM; (**E**) The correlation between CXCL2 gene expression and TAN related risk score.

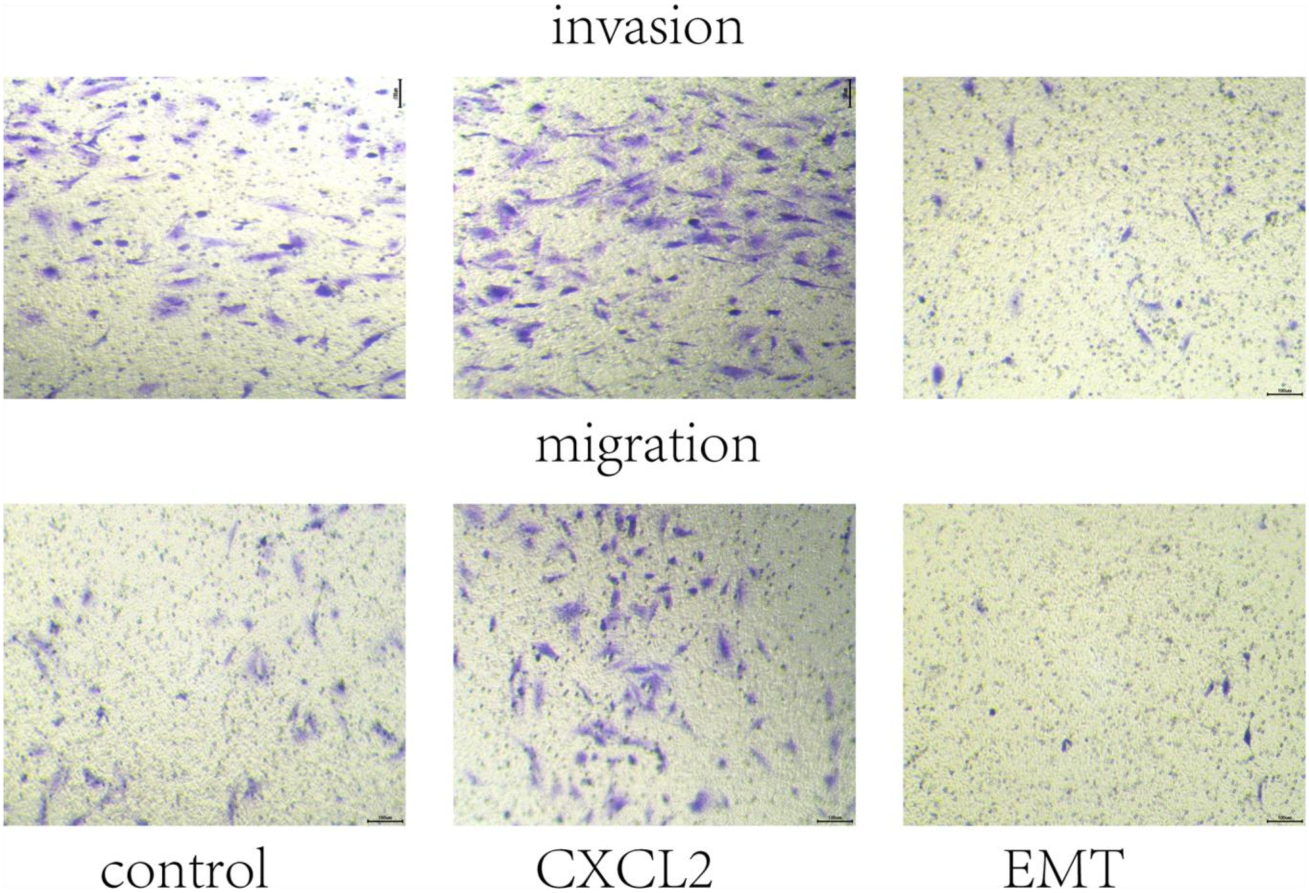

Supplementary Figure 2. The cell invasion and migration abilities under three conditions: control group, the CXCL2 (100 ng/ml) group, and CXCL2 (100 ng/ml) combined with EMT inhibitor 1 group.
