# Supplementary material for: Prognostic significance and mechanisms of CXCL genes in clear cell renal cell carcinoma
Source: Aging (Albany NY). 2023 Aug 3;15(16):7974–96. doi: 10.18632/aging.204922 (PMC10497021; doi:10.18632/aging.204922)
Supplement: Supplementary Table 1 [file aging-15-204922-s003.pdf]

SUPPLEMENTARY TABLE

Supplementary Table 1. The markers from two TAN subtypes (N2 subtype was pro-tumoral and N1 subtype was anti-tumoral) from the literature.

TAN 1 marker	TAN 2 marker
ARG1	ARG1
CCL3	IL-6
CXCL10	IL-17
CXCL9	IL-8
H2O2	CXCL5
IFNB1	G-CSF
IL-1B	TGFB1
IL-8	GM-CSF
NETS	TNF
ROS	MPO
TNF	CXCL2
	CCL2
	CCL5
	CCL3
	CCL17
	ROS
	NE
	VEGF
	H2O2
	MMP9
	HGF
	OSM
	NETS
